# Supplementary material for: Novel Driver Strength Index highlights important cancer genes in TCGA PanCanAtlas patients
Source: PeerJ. 2022 Aug 11;10:e13860. doi: 10.7717/peerj.13860 (PMC9375969; doi:10.7717/peerj.13860)
Supplement: Supplemental Information 2 [file peerj-10-13860-s002.zip › COHORTS/genes plots/detailed/2021_8_16_14_9_distribution_events_detailed_36.pdf]

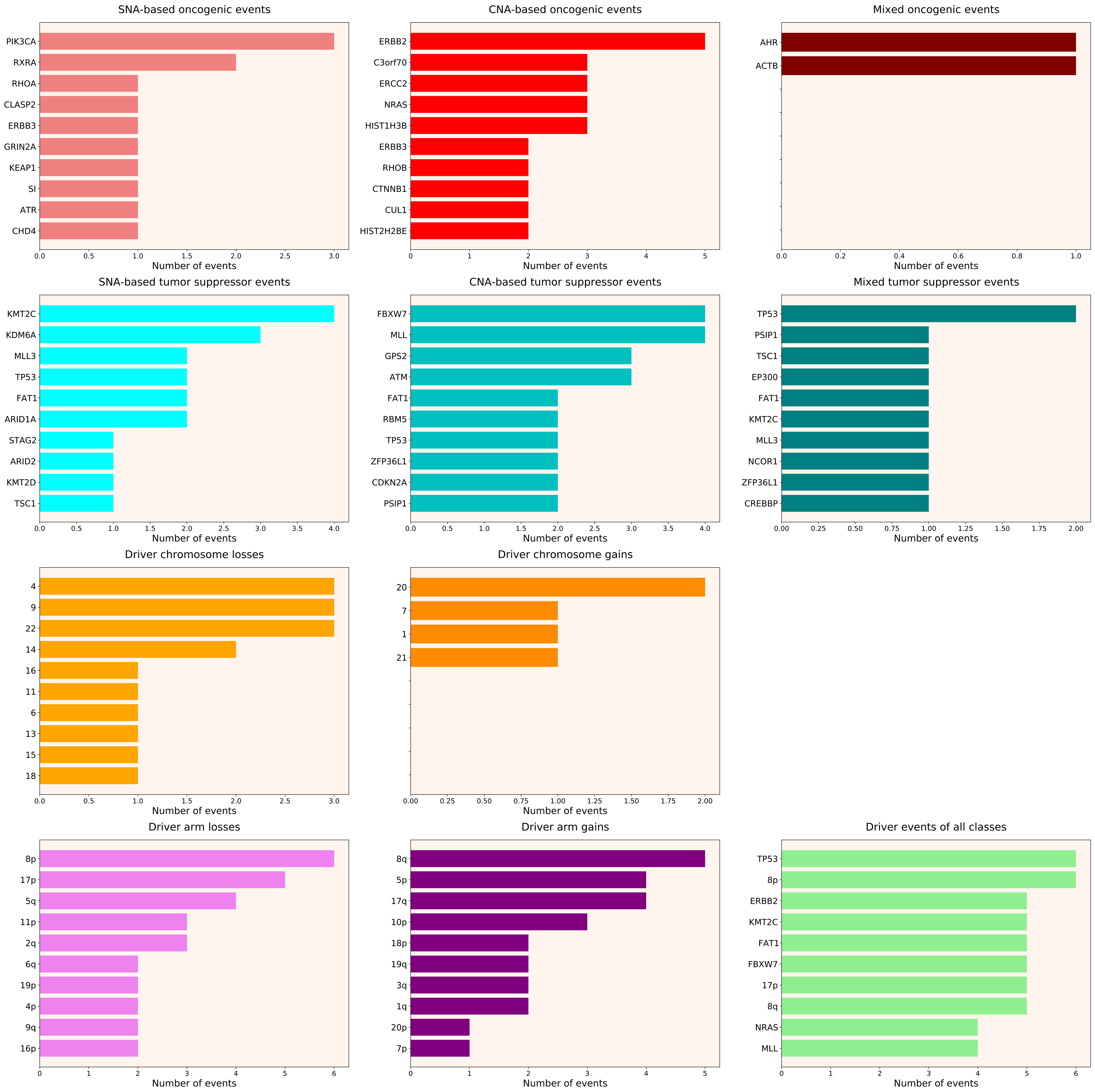
SNA-based oncogenic events
PIK3CA
RXRA
RHOA
CLASP2
ERBB3
GRIN2A
KEAP1
SI
ATR
CHD4
0.0 0.5 1.0 1.5 2.0 2.5 3.0
Number of events
CNA-based oncogenic events
ERBB2
C3orf70
ERCC2
NRAS
HIST1H3B
ERBB3
RHOB
CTNNB1
CUL1
HIST2H2BE
0 1 2 3 4 5
Number of events
Mixed oncogenic events
AHR
ACTB
0.0 0.2 0.4 0.6 0.8 1.0
Number of events
SNA-based tumor suppressor events
KMT2C
KDM6A
MLL3
TP53
FAT1
ARID1A
STAG2
ARID2
KMT2D
TSC1
0.0 0.5 1.0 1.5 2.0 2.5 3.0 3.5 4.0
Number of events
CNA-based tumor suppressor events
FBXW7
MLL
GPS2
ATM
FAT1
RBM5
TP53
ZFP36L1
CDKN2A
PSIP1
0.0 0.5 1.0 1.5 2.0 2.5 3.0 3.5 4.0
Number of events
Mixed tumor suppressor events
TP53
PSIP1
TSC1
EP300
FAT1
KMT2C
MLL3
NCOR1
ZFP36L1
CREBBP
0.00 0.25 0.50 0.75 1.00 1.25 1.50 1.75 2.00
Number of events
Driver chromosome losses
4
9
22
14
16
11
6
13
15
18
0.0 0.5 1.0 1.5 2.0 2.5 3.0
Number of events
Driver chromosome gains
20
7
1
21
0.00 0.25 0.50 0.75 1.00 1.25 1.50 1.75 2.00
Number of events
Driver arm losses
8p
17p
5q
11p
2q
6q
19p
4p
9q
16p
0 1 2 3 4 5 6
Number of events
Driver arm gains
8q
5p
17q
10p
18p
19q
3q
1q
20p
7p
0 1 2 3 4 5
Number of events
Driver events of all classes
TP53
8p
ERBB2
KMT2C
FAT1
FBXW7
17p
8q
NRAS
MLL
0 1 2 3 4 5 6
Number of events
